# Assessing the Uses, Benefits, and Limitations of Digital Technologies Used by Health Professionals in Supporting Obesity and Mental Health Communication: Scoping Review

**DOI:** 10.2196/58434

**Published:** 2025-02-10

**Authors:** Amanda Kearns, Anne Moorhead, Maurice Mulvenna, Raymond Bond

**Affiliations:** 1 Life and Health Sciences Institute for Nursing and Health Research Ulster University Belfast United Kingdom; 2 School of Communication and Media Institute for Nursing and Health Research Ulster University Belfast United Kingdom; 3 School of Computing Ulster University Belfast United Kingdom

**Keywords:** digital communication, digital technology, digital transformation, health professional, mental health, obesity, complex needs, artificial intelligence, AI, PRISMA

## Abstract

**Background:**

Obesity and mental health issues present interconnected public health challenges that impair physical, social, and mental well-being. Digital technologies offer potential for enhancing health care communication between health professionals (HPs) and individuals living with obesity and mental health issues, but their effectiveness is not fully understood.

**Objective:**

This scoping review aims to identify and understand the different types of technologies used by HPs in supporting obesity and mental health communication.

**Methods:**

A comprehensive scoping review, which followed a validated methodology, analyzed studies published between 2013 and 2023 across 8 databases. The data extraction focused on HPs’ use of communication technologies, intervention types, biopsychosocial considerations, and perceptions of technology use. The review was guided by the following research question: “What are the uses, benefits, and limitations of digital technologies in supporting communication between HPs and persons living with obesity and mental health issues?”

**Results:**

In total, 8 studies—featuring web-based platforms, social media, synchronous video calls, telephone calls, automated SMS text messaging, and email—met the inclusion criteria. Technologies such as virtual learning collaborative dashboards and videoconferencing, supported by automated SMS text messaging and social media (Facebook and WhatsApp groups), were commonly used. Psychologists, dietitians, social workers, and health coaches used digital tools to facilitate virtual appointments, diet and mental health monitoring, and motivational and educational support through group therapy, 1-on-1 sessions, and hybrid models. Benefits included enhanced access to care and engagement, personalized digital cognitive behavioral therapy, perceived stigma reduction, privacy, and improved physical health outcomes in weight reduction. However, improvements in mental health outcomes were not statistically significant in studies reporting *P* values (*P*≥.05). The limitations included engagement difficulties due to conflicting personal family and work commitments; variable communication mode preferences, with some preferring in-person sessions; and misinterpretations of SMS text messaging prompts. Conflicts arose from cultural and individual differences, weight stigma, and confusion over HP roles in obesity and mental health care.

**Conclusions:**

Digital technologies have diversified the approaches HPs can take in delivering education, counseling, and motivation to individuals with obesity and mental health issues, facilitating private, stigma-reduced environments for personalized care. While the interventions were effective in obesity management, the review revealed a shortfall in addressing mental health needs. This highlights an urgent need for digital tools to serve as media for a deeper engagement with individuals’ complex biopsychosocial needs. The integration of data science and technological advancements offers promising avenues for tailored digital solutions. The findings advocate the importance of continued innovation and adaptation in digital health care communication strategies, with clearer HP roles and an interdisciplinary, empathetic approach focused on individual needs.

## Introduction

### Background

Obesity and mental health issues, two of the greatest global public health challenges [1], are intricately linked in a bidirectional and complex relationship that impacts both physical and mental well-being [2]. People experiencing depression are found to have a 58% increased risk of becoming obese [3]. Conversely, obesity is associated not only with physiological challenges but also social and mental health burdens [4], with persons who are obese facing an increased risk of developing depression over time. This relationship is particularly pronounced among people with serious mental illnesses (SMIs) such as schizophrenia or bipolar disorders, where obesity prevalence ranges between 45% and 55% [5]. Mental disorders account for the leading cause of global disability and determine how individuals make healthy choices [6], impacting personal well-being and quality of life [7]. The intertwined nature of obesity and mental health problems has a substantial impact on socioeconomic and health care systems [7]. The increasing prevalence of these conditions in society [8] calls for an urgent need to address this dual challenge to improve global health outcomes.

Health professionals (HPs) play an integral role in communicating mental health and obesity risks to the public [9]. Effective communication has been shown to positively impact the relationship between the HP and service users, engagement, and patient outcomes [10]. Communication regarding weight and mental health is especially challenging because communication training for HPs focuses on the medical aspects rather than the complexities of the conditions [11]. HPs require appropriate guidelines to identify users at risk of mental health issues [12]. Technology advances have the potential to transform health care, as evidenced by their critical role in communicating health messages during the COVID-19 pandemic [13]. According to Thomas [14], health communication is defined as “the systematic study and practical application of communication strategies in health, to inform and influence the attitudes, knowledge, and practices of individuals and communities.” Within this domain, the co-occurrence of obesity and mental health issues presents a challenge. While there is an increasing acknowledgment of the importance of these interrelated health issues, a distinct gap exists in the literature. Currently, there is no review that addresses the intersection of mental health issues and obesity.

The rates of coexisting obesity and mental health issues are rising [15,16] owing to a multitude of physical, psychological, and environmental factors, such as medications, unhealthy diet, alcohol misuse, sedentary lifestyles, and low mood affecting adherence to weight management (WM) interventions [3,17-19]. Despite this growing concern, research studies often overlook the interplay between obesity and mental health issues, particularly in how these conditions are addressed and communicated by HPs. Current multidisciplinary guidelines for HPs dealing with obesity and mental illness recommend that they monitor and address patients’ lifestyles; yet, research has found that most HPs have limited lifestyle-related knowledge and skills and that lifestyle treatment protocols are lacking [16]. There is a lack of understanding among HPs of how to integrate digital health solutions into everyday practices and procedures [20]. Existing research shows a need for obesity and mental health communication support for HPs. Obesity and mental health issues are acknowledged as a global health priority, but medical and sociocultural characteristics of the population are not taken into consideration [21]. Thus, research is required to explore the use of digital communication aids among HPs and the growing challenges presented by aging populations with multimorbidities.

### Objectives

This scoping review aims to identify and understand the different types of technologies that are used by HPs in supporting obesity and mental health communication. This scoping review was guided by the following broad central research question: “What are the uses, benefits, and limitations of digital technologies in supporting communication between HPs and persons living with obesity and mental health issues?”

## Methods

### Scoping Review Framework

A scoping review applying a systematic approach was conducted as per the guidance of Munn et al [22] on choosing between different approaches when conducting evidence synthesis. This novel method was selected to address the broad research question guiding this review: to scope the literature for knowledge gaps [23] and gain conceptual clarity on the adoption of digital technologies in health care communication by thematically analyzing the available literature [24]. The framework developed by Arksey and O’Malley [25] guided this review, as recommended by Levac et al [23] and Daudt et al [26]. A scoping review applying a systematic approach was conducted, as per the guidance of Munn et al [22] on choosing between different approaches when conducting evidence synthesis, and the Cochrane Handbook for Systematic Reviews of Interventions [27] for systematic review methodology. This involved the following steps: (1) identifying the research question, (2) identifying relevant studies, (3) study selection, (4) charting the data, and (5) summarizing and reporting the results [25]. The review process applied the rigorous standards of the PRISMA-ScR (Preferred Reporting Items for Systematic Reviews and Meta-Analyses extension for Scoping Reviews) checklist [28] (Multimedia Appendix 1) as the latest and most advanced approach for transparent and comprehensive reporting of scoping reviews.

### Research Question

The purpose of this scoping review is to present the results of existing literature addressing the role of digital technologies used by HPs in facilitating communication with individuals living with obesity and mental health issues. The guiding research question was as follows: “What are the uses, benefits, and limitations of digital technologies in supporting communication between HPs and persons living with obesity and mental health issues?”

### Search Strategy

The inclusion criteria followed the JBI scoping review guide [29], using *population*, *concept*, and *context*. The review included studies of all designs to identify the best evidence available to address the research objective. To support a thorough search, no time frame limit was applied, and all languages were included if translation was possible using Google Translate [30].

Eight electronic databases—Ovid MEDLINE, CINAHL, Scopus, ScienceDirect, PsycINFO, IEEE Xplore, ACM Digital Library, and ClinicalTrials.gov—were systematically searched in April 2023. These databases were chosen to capture articles in health care, psychology, and computer science fields. In addition, manual citation searching was performed to identify any relevant sources that might have been overlooked by the database searches.

Due to the novelty of the topic concerned, a broad search strategy was developed with high sensitivity to the following key terms: (1) “health professional,” (2) “digital technology,” (3) “obesity,” and (4) “mental health.” On the basis of the literature, benchmark definitions for these concepts were developed. A total of 144 synonyms were formulated for the search strings (Multimedia Appendix 2) with the guidance of a trained librarian. From the database searches and manual citation searching, 1656 hits were identified (Multimedia Appendix 3).

### Study Selection

Following the framework outlined by Arksey and O’Malley [25] and aligning with the exploratory nature of scoping reviews, this study included primary research articles on digital technology interventions supporting HP communication with individuals facing obesity and mental health challenges. Exclusions were made for sources not directly related to HP communication support, such as those focused on technology use during HP training, which were deemed to be outside the scope of this review. In addition, conference papers, design protocols, and abstracts without full studies were excluded. The full eligibility criteria are presented in Textbox 1.

**Eligibility criteria for the scoping review.**

Inclusion criteria

- Population: health professionals (HPs) and clients living with obesity and mental health issues
- Concept: studies that implement digital technology interventions aimed at enhancing communication processes
- Context: comorbidities of obesity and mental health issues
- Study characteristics: primary research studies; no time limits applied; all languages included if translation possible using Google Translate

Exclusion criteria

- Population: intervention without a focus on HP communication support
- Concept: digital technology intervention for clients with obesity and mental health issues, with no clear supporting role of HPs
- Context: broad or general comorbidities, not including obesity and mental health issues specifically
- Study characteristics: conference papers and design protocols

Articles identified from the systematic search were managed using the Covidence software platform (Veritas Health Innovation Ltd) [31]. Titles and abstracts were screened by 2 reviewers (AK and AM) independently (n=1078). The full text of relevant articles identified during the title and abstract screening was examined for eligibility against the inclusion and exclusion criteria by 2 independent reviewers (AK and AM). Ultimately, of the 1078 articles screened, 8 (0.74%) studies exemplifying the use of technology in HP communication for obesity and mental health issues were selected for analysis.

### Charting and Extracting Data

Adhering to the JBI guidelines, a tailored data extraction template focused on study details, participant characteristics, interventions, and outcomes. The Covidence template was checked for suitability by 2 reviewers (AM and MM) before data extraction commenced. One author (AK) extracted data, which were verified by a second author (AM); disagreements were resolved by discussion. A thematic analysis identified key concepts related to HP communication technologies, including types of technology use, HP roles, biopsychosocial considerations, and perceptions regarding benefits and limitations in obesity and mental health contexts.

### Summarizing and Reporting Results

The data charting process identified key themes, which were mapped around the biopsychosocial theoretical model developed by Engel [32] to facilitate an understanding of the results. Data synthesis involved comparing methodologies and results across the 8 studies to identify trends and gaps.

### Quality Appraisal

The primary aim of the scoping review was to map out the diversity of technology use in HP communication; however, to enhance the reliability of the findings on technology use in HP communication for obesity and mental health issues, a detailed quality appraisal of the included studies was conducted. This involved using the revised Cochrane risk-of-bias tool for randomized trials (RoB 2) for randomized controlled trials (RCTs) [33] and the Risk of Bias in Nonrandomized Studies of Interventions (The Cochrane Collaboration) tool for nonrandomized studies [34]. This dual approach helped critically evaluate the evidence base, identifying studies with lower risks of bias that could provide more dependable insights into the effectiveness of digital technology interventions for managing obesity and mental health issues.

Eight original research studies integrating technology for HP communication support were critiqued for study design, implementation, and reporting [35-42]. The studies were rigorously assessed by 2 independent reviewers (AK and AM), using a grading key for risk of bias (ROB): (1) low ROB, (2) some concerns, and (3) high ROB.

## Results

### Selected Studies

**Figure 1:**
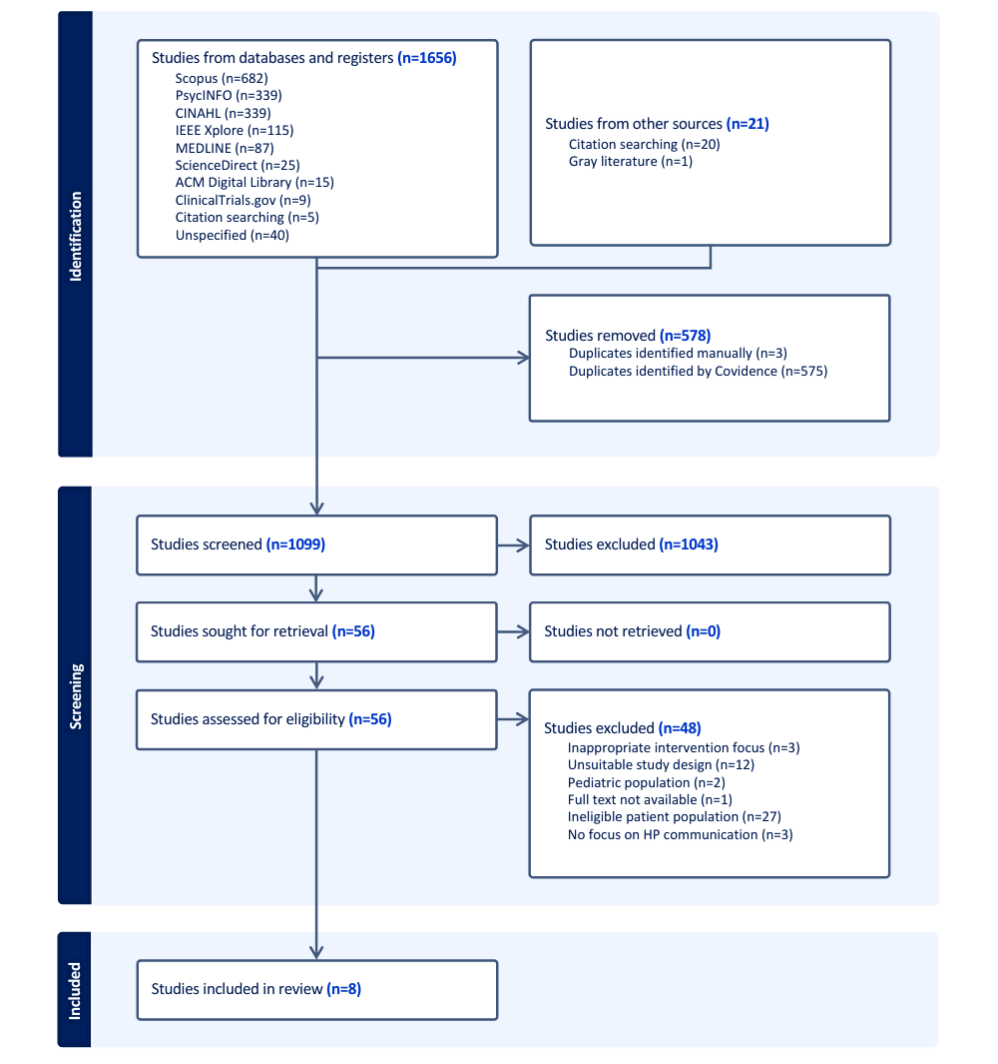
PRISMA-ScR (Preferred Reporting Items for Systematic Reviews and Meta-Analyses extension for Scoping Reviews) flow diagram outlining the literature search and study selection procedures. HP: health professional.

This scoping review included 8 studies: 4 (50%) RCTs, 1 (12%) pilot RCT, and 3 (38%) qualitative feasibility studies. They were conducted in the United States (5/8, 62%), the United Kingdom (1/8, 12%), South Korea (1/8, 12%), and Iran (1/8, 12%; the percentages do not add up to 100% due to rounding). The study selection process is detailed in the PRISMA-ScR flow diagram (Figure 1), outlining the inclusion and exclusion steps taken during the literature review.

Key data from each study, including publication year, country, design, sample size, aim, context, demographic details, key findings, and limitations, were systematically charted (Table 1).

**Table 1:** Data extraction charting for the included scoping review studies (n=8)^a^.

Study; year; country; study design	Aim	Participants, n; context; sex: %; age (y)	Key findings and conclusions	Comments
Lee et al [35]; 2023; United Kingdom; qualitative feasibility	Enhance web-based WM^b^ program uptake for SMI^c^ using VLC^d^	17; web-based, participants with SMI; female: 76; ≥18 (mean 48, SD not reported; range 29-70)	Promising engagement through digitally enhanced augmented WM program, complemented by brief, targeted education and low-intensity check-ins	Small size; potential bias
Bartels et al [38]; 2022; United States; hybrid RCT^e^	Compare web-based VLC vs TA^f^ in the InSHAPE health promotion program for individuals with SMI	HPs^g^: 55; CMHC^h^, with patients with SMI: 1219; female: % not reported; ≥18 (mean and SD not reported)	VLC as effective as TA in SMI obesity management	Sample representativeness; no cost data
Aschbrenner et al [36]; 2022; United States; 2-arm RCT	Evaluate PeerFIT mHealth^i^ online Facebook group vs 1-on-1 mHealth (BEAT^j^) coaching for young adults with SMI who were overweight or obese	150; CMHC, young adults with SMI; female: 55; 18-35 (mean 28.46, SD 4.5)	PeerFIT and mHealth coaching equally effective, indicating that low-intensity mHealth coaching is a scalable, beneficial intervention option	Blinding issues; COVID-19 impact on retention; low engagement; lack of text or activity tracker data access; unexplored intervention format preferences
Nicol et al [41]; 2022; United States; qualitative feasibility	Test web-based iOTA^k^ method (SMS text messaging and in-person coaching) for adults with SMI undergoing obesity treatment	26; CMHC, adults with SMI; female: 60; 16-75 (mean 48.5, SD 15.67)	iOTA method feasible; engagement varied; outcomes influenced by symptom severity, indicating a need for tailored adaptations	Small heterogeneous sample; implementation challenges; more comprehensive symptom assessments needed
Haddad et al [39]; 2022; United States; qualitative feasibility	Adapt mHealth obesity treatment for adults with SMI using web-based SMS text messaging I-Corps^l^ methods	29; CMHC, various stakeholders, participants with SMI unspecified; sex not reported; age not reported	Feasibility of adapted mHealth obesity treatment; stakeholder insights: need for microadjustments in clinical settings and clarity in health coaching responsibilities	Limited consumer input; reporting bias; no specific differentiation in SMI diagnosis
Abedishargh et al [37]; 2021; Iran; 3-arm RCT	Assess ICBT^m^ on WhatsApp for mental health issues of women with overweight	90; online support group, participants with depression or anxiety; female: 100; 18-50, 38.2% 18-30 (mean and SD not reported)	ICBT reduced BMI but not mental health issues	No follow-up; pandemic influence on short duration of therapy; online implementation challenges; sample selection process not detailed
Yu et al [42]; 2021; United States; pilot RCT	Evaluate videoconferencing-based treatment for BED^n^	18; web-based, participants with obesity with BED; female: 100; 20-73 (mean 32, SD 15.27)	Videoconferencing-based treatment improved shape concern but not weight	Small size; high dropout rate; technical issues; potential self-reported bias
Kim et al [40]; 2020; South Korea; 2-arm RCT	Test digital CBT^o^ for WM in adults with depression or anxiety using intensive, multifactorial, daily psychologist coaching	70; online, participants with depression or anxiety; female: 100; 18-39 (mean 21.8, SD 3.3)	Effective CBT integration in digital WM combined with multidisciplinary human support	Generalizability; self-report inaccuracies; feasibility: intensive and costly, requiring daily intervention by trained therapists

^a^Summary: study design: 4 randomized controlled trials (RCTs), 1 pilot RCT, and 3 qualitative feasibility studies; conducted in the United States (5/8, 62%); aim: assessed digital interventions including web-based virtual learning collaborative dashboards, SMS text messaging, and online support groups for improving engagement and effectiveness in serious mental illness and obesity; participants: n=17–150; contexts included community mental health centers (50%) and online communities (50%); primarily female, 16–75 years (mean 35.8, SD 9.68; % female unknown in some studies); key findings: interventions improved engagement and effectiveness but faced limitations like small samples, biases, and generalizability issues; comments: limitations included small sample sizes, biases, and feasibility concerns.

^b^WM: weight management.

^c^SMI: serious mental illness.

^d^VLC: virtual learning collaborative.

^e^RCT: randomized controlled trial.

^f^TA: technical assistance.

^g^HP: health professional.

^h^CMHC: community mental health center.

^i^mHealth: mobile health.

^j^BEAT: Basic Education Supported by Activity Tracking.

^k^iOTA: interactive obesity treatment approach.

^l^I-Corps: Innovation Corps.

^m^ICBT: internet-based cognitive behavioral therapy.

^n^BED: binge eating disorder.

^o^CBT: cognitive behavioral therapy.

### Participant Characteristics

The participant pool varied, ranging from 17 to 150 participants per study. Half of the studies (4/8, 50%) were conducted at community mental health centers [36,38,39,41], and the other half (4/8, 50%) involved web-based recruitment of the general population [35,37,40,42]. Most participants were female, with sex data reported in 75% (6/8) of studies (range 55%-100%). Recruitment ages ranged from 16 to 75 years, with a mean age of 35.8 years. Among the 4 studies that reported SDs (50%), the average SD was 9.68 years (Table 1). The studies used various tools to assess mental health conditions such as SMI, depression, anxiety, and binge eating disorder, providing a diverse insight into mental health evaluations in the context of digital technology use in health care (Table 2).

**Table 2:** Participant mental health (MH) characteristics and assessment tools in the included studies (n=8).

Participant criteria for MH issues	Studies, n (%)	Specific MH assessment tools	Tools used (n=14), n (%)
SMI^a^ (as defined by, eg, schizophrenia, schizophreniform disorder, schizoaffective disorder, bipolar disorder, or depression with psychosis) [41]	1 (12)	Clinical Global Impression severity score of >5	1 (7)
Depression, anxiety (with stress or self-esteem issues) [37,40]	2 (25)	Situational Motivation Scale Korean version of the Beck Depression Inventory-II Taylor Anxiety Inventory Rosenberg Self-Esteem Scale Automatic Thoughts Questionnaire-30 Depression Anxiety Stress Scales	6 (43)
BED^b^ [42]	1 (12)	Eating Disorder Examination Questionnaire 6.0 Eating Attitudes Test-26 Three-Factor Eating Questionnaire–Revised 18 Yale Food Addiction Scale Symptom Checklist-90–Revised Beck Depression Inventory-II Impact of Weight on Quality of Life–Lite	7 (50)
SMI but no specified assessment tools, that is, no specificity of MH issues [35,39] or measured fidelity and cardiovascular risk reduction only [36,38]	4 (50)	None	0 (0)

^a^SMI: serious mental illness.

^b^BED: binge eating disorder.

### Uses of Digital Technologies in Obesity and Mental Health Communication

**Figure 2:**
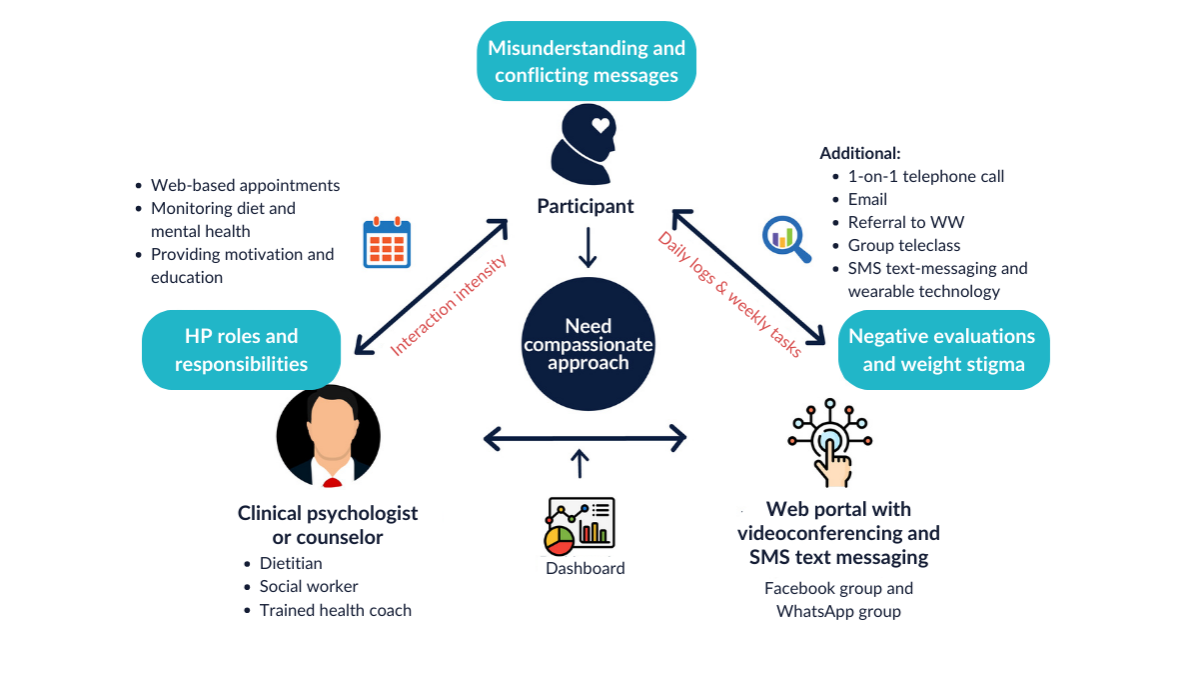
Mapping of the uses of digital technologies and key themes from the 8 included studies [35-42] regarding the role of digital technologies in supporting communication between health professionals (HPs) and participants with obesity and mental health issues. WW: WeightWatchers.

The investigation into the use of digital technologies across the 8 included studies highlights their role in addressing obesity and mental health issues. These technologies facilitated virtual appointments, diet and mental health monitoring, and the provision of motivational and educational content. In terms of professional use, psychologists or counselors were consistent users across all studies [35-42], with additional involvement from dietitians [38,42], social workers [36,39], and trained health coaches [35,38] (Figure 2 [35-42]).

The primary digital interventions varied. They included web-based platforms with synchronous video calls (4/8, 50%), automated SMS text messaging (2/8, 25%), and social media (Facebook and WhatsApp groups; 2/8, 25%). Various supplementary support methods were used, including 1-on-1 telephone calls (3/8, 38%), email (3/8, 38%), group teleclasses (2/8, 25%) [37,42], referrals to WeightWatchers (2/8, 25%), and the integration of SMS text messaging with wearable technology (1/8, 12%). The most frequently reported tool was the virtual learning collaborative (VLC) dashboard with its videoconferencing feature [35,38,40,42]. The most common supplementary support methods used were 1-on-1 telephone calls [35,38,40] and email [35,38,42].

The 8 studies illustrated diversity in their approach to digital interventions, with 4 (50%) focusing on group therapy, 2 (25%) on 1-on-1 interventions, and 2 (25%) using a hybrid model that combined both methods. HP interaction frequencies varied, with the most common being daily interactions (3/8, 38%). Typical session durations were either 1 hour (2/8, 25%) or 1.5 hours (2/8, 25%), and the interventions spanned an average duration of 3 months (3/8, 38%, SD 1.63 months), with the total number of sessions ranging from 4 to 52 sessions with an HP (Table 3).

**Table 3:** Data extraction results for the included studies on digital interventions and health professional (HP) communication (n=8)^a^.

Study; year		Intervention name	Secondary support	HPs involved	Contact time	Interaction	Duration (mo)	Sessions, n	Adherence	Theory
**Main digital intervention: web portal VLC^b^ dashboard with videoconferencing**										
	Lee et al [35]; 2023	WHEEL^c^; Meet Your Mentor (single 1.5-h, web-based, 1-on-1 consultation)	Telephone call or email mentor check-ins plus referral to WW^d^ group	Psychologist	90-min plus 15-min interim booster support	Weekly (fixed day and time) for 12 wk	3	12	Good	PBA^e^ (based on the MRC^f^ model)
	Bartels et al [38]; 2022	InSHAPE VLC	Telephone call or email mentor check-ins plus referral to WW group	Trained fitness instructor receives instruction from health psychologist, dietitian, and senior-level InSHAPE program manager and trainer	4-d InSHAPE training plus 90-min follow-up sessions	Monthly	12-18	50	Good	VLC
	Yu et al [42]; 2021	Fruit Street videoconferencing program	Group teleclass and email	Psychologist and dietitian	1 h	Weekly	3	12	Good	CBT^g^
	Kim et al [40]; 2020	Noom coach and InBody	1-on-1 telephone calls	Psychologist	Weekly group call plus 15-min interim booster support	Daily	2	8	Good	CBT+MI^h^
**Main digital intervention: SMS text messaging**										
	Haddad et al [39]; 2022	Innovation Corps iOTA^i^	—^j^	Psychologist, prescribing physician, nurse, and social worker	—	—	—	—	Not reported	VLC
	Nicol et al [41]; 2022	Working for You iOTA	—	Trained health coach at mental health clubhouse; no specific roles	—	Monthly	3	4	Adequate	Chronic care model
**Main digital intervention: Facebook or WhatsApp groups**										
	Aschbrenner et al [36]; 2022	PeerFIT	SMS text messaging and wearable technology	Psychologist, counselor, social worker, or fitness professionals	1 h	Daily	12	52	Poor	SCT^k^+DPP^l^
	Abedishargh et al [37]; 2021	Weight loss	Group teleclass	Psychologist plus peer-led interventions	2 h	Daily	1.5	42	Good	CBT

^a^Table 3 summarizes various digital interventions (web portals, virtual learning collaborative [VLC], SMS text messaging, and social media), with mixed delivery methods (1-on-1 telephone calls, mentor check-ins, and group classes) and diverse health professionals’ involvement (psychologists, dietitians, social workers, and fitness instructors). Contact time ranged from 15-minute booster sessions to 90-minute consultations, with interaction frequencies varying from daily to monthly. Intervention durations ranged from 1.5 to 18 months, with 4 to 52 sessions. Adherence varied, and theories included cognitive behavioral therapy, VLC, motivational interviewing, social cognitive theory, Diabetes Prevention Program, and person-based approach.

^b^VLC: virtual learning collaborative.

^c^WHEEL: Weight Change for People With Serious Mental Illness.

^d^WW: WeightWatchers.

^e^PBA: person-based approach.

^f^MRC: Medical Research Council.

^g^CBT: cognitive behavioral therapy.

^h^MI: motivational interviewing.

^i^iOTA: interactive obesity treatment approach.

^j^Not applicable.

^k^SCT: social cognitive theory.

^l^DPP: Diabetes Prevention Program.

Content-wise, the digital interventions drew upon a range of theoretical frameworks and strategies, including cognitive behavioral therapy (CBT; 2/8, 25%), CBT plus motivational interviewing (1/8, 12%), VLC (2/8, 25%), the person-based approach following the Medical Research Council model (1/8, 12%), the chronic care model (1/8, 12%), and the social cognitive theory supplemented with Diabetes Prevention Program content (1/8, 12%; Table 3).

### Quality Appraisal

The studies varied in quality. Of the 5 RCT studies, 2 (40%) were rated as high quality with low ROB [38,40], and 3 (60%) were deemed to have an acceptable ROB [36,37,42] based on the ROB 2 (Table 4), while the qualitative feasibility studies were consistently rated as high quality and low ROB [35,39,41] based on the Risk of Bias in Nonrandomized Studies of Interventions tool (Table 5).

**Table 4:** Quality assessment of 5 included randomized controlled trial studies using the Cochrane risk-of-bias tool for randomized trials (ROB 2)^a^.

Study; year	Risk of bias: methods				Risk of bias: results			Overall risk of bias
	Study design	Participants	Assessments and data collection	Reporting quality		Clinical efficacy		
Bartels et al [38]; 2022	++^b^	++	++	++		++	++	
Aschbrenner et al [36]; 2022	++	+^c^	+	+		+	+	
Abedishargh et al [37]; 2021	++	++	+	+		+	+	
Yu et al [42]; 2021	++	+	+	+		+	+	
Kim et al [40]; 2020	++	++	++	++		++	++	

^a^The studies demonstrated a low risk of bias in methods and results overall, though some concerns were noted in assessments, reporting quality, and clinical efficacy. Variability in risk levels was observed across study design and participant selection

^b^++: high quality and low risk of bias.

^c^+: acceptable risk of bias.

**Table 5:** Quality assessment of 3 included qualitative feasibility studies using the Risk of Bias in Nonrandomized Studies of Interventions (ROBINS-I) tool^a^.

Study; year	Risk of bias						
	Confounding	Participant selection	Intervention classification	Deviations from intended interventions	Missing data	Measurement of outcomes	Selection of reported result
Lee et al [35]; 2023	Low	Low	Low	Low	Low	Moderate	Low
Nicol et al [41]; 2022	Moderate	Low	Low	Moderate	Low	Moderate	Low
Haddad et al [39]; 2022	Moderate	Low	Low	Moderate	Low	Low	Low

^a^Across the studies, deviations from intended interventions were assessed as moderate risk of bias, while low risk of bias was consistently noted for participant selection, intervention classification, missing data, and selection of reported results. Risk of bias for outcome measurement was moderate in 67% (2/3) of studies.

### Benefits of Digital Technologies in Obesity and Mental Health Communication

The digital interventions provide benefits such as improved access to personalized care via digital CBT, tailored feedback, and enhanced physical health outcomes, including weight loss and BMI improvement. The study by Haddad et al [39] unveiled that some service users had previous negative in-person interactions with HPs, perceiving limited benefit relative to the “additional burden of time and mental energy” invested, and some participants preferred online intervention delivery because it helped reduce stigma (Multimedia Appendix 4 [35-42]). The digital format offered a supportive and private environment conducive to client adherence, motivation, engagement, and comfort, thereby reducing the stigma associated with seeking help [36,37]. Participants with mood disorders reported experiences of shame and negative self-evaluation [33], showing the importance of addressing self-esteem issues within digital health platforms. Personalization and engagement emerged as key factors in the success of digital interventions [39]. Programs with digital CBT, adapted to individual characteristics and supported by intensive daily monitoring, were shown to improve participant engagement and adherence with minimal dropout rates: as noted by Bartels et al [38], “only one drop out for personal reasons, and 80% remained active until the end of the treatment session. Authors owe this to motivation construct.”

Efforts to enhance the implementation and effectiveness of digital interventions include comprehensive training for health mentors, involving psychologists, dietitians, and program managers [37]. This indicates the significance of a multidisciplinary approach and the need for sustainable funding and training solutions: and training solutions. For instance, Abedishargh et al [37] highlighted the challenge of “finding a funding source to pay for a dietitian,” while Bartels et al [38] emphasized the importance of “training changes and sustainability of a wellness program.” The collaboration within VLC programs, intensive skill-building training, and the involvement of mentors and leadership emphasize the value of mentorship and a compassionate approach in program delivery [36]. Significant improvements in WM were observed in 7 (88%) of the 8 studies [35-41], showing the efficacy of digital interventions in supporting better physical health outcomes.

The fact that there was no significant improvement in mental health outcomes suggests a potential inadequacy in the ability of the digital interventions to simultaneously address mental health needs and obesity. Yu et al [42] observed no significant changes in weight and binge eating episodes among participants. Conversely, Nicol et al [41] demonstrated that individuals with less severe symptoms and higher engagement achieved statistically significant weight loss (*P*<.001) compared to those with more severe symptoms. Nonetheless, clarity regarding the inclusion criteria for individuals with SMI was lacking in the studies, and there was inconsistency in the measurement of outcomes related to mental health status (Table 2). These findings indicate a need for further research that adopts a comprehensive and multidisciplinary approach to digital health interventions.

### Limitations of Digital Technologies in Obesity and Mental Health Communication

Challenges in implementing digital technologies include misinterpretations of SMS text messaging prompts [41], variability in user engagement, and adherence [35,36,38,42]. These challenges highlight the importance of flexible and adaptable interventions, personalized to individual lifestyles and preferences, and a clear understanding of HP roles in digital interventions. The recurrent call for dietetic expertise [38-40,42] identifies a gap in incorporating comprehensive nutritional guidance within these interventions, advocating for a multidisciplinary strategy that includes dietitians to offer balanced and scientifically sound dietary advice tailored to the needs of individuals managing obesity and mental health issues (Table 6).

**Table 6:** Key further research questions identified from the scoping review.

Research question	Relevance	Directions for future research
How can digital interventions be tailored to better address the mental health needs of individuals with comorbid obesity and mental health issues?	Psychosocial	Investigate adaptive features and content within digital interventions to provide comprehensive mental health support alongside obesity management.
How can personalized care be enhanced in digital interventions for obesity and mental health care?	Psychosocial	Examine methods for deep personalization in digital interventions, considering the complexity of comorbid conditions and individual user needs, focusing on unique needs, engagement, and outcomes.
What are the optimal strategies for enhancing user engagement and adherence to digital interventions?	Psychosocial	Develop and test engagement strategies that account for individual preferences for, and barriers to, sustained use of digital health tools, while incorporating robust adherence measures.
How can digital interventions be designed to support a more compassionate and empathetic approach to obesity and mental health care?	Psychosocial	Explore the integration of features that enhance empathy, compassion, and effective communication between clients and HPs^a^ in digital health platforms, promoting patient satisfaction, trust, and improved outcomes.
What are the optimal roles and responsibilities of HPs in digital interventions to ensure high fidelity and participant retention?	Social	Determine the best practices for multidisciplinary collaboration in digital health interventions, optimizing HP roles, involvement, and training needs.

^a^HP: health professional.

Variability in intervention intensity and the roles of HPs present challenges in achieving effective program outcomes. The comparison between a nutritionist-led “treatment as usual” group and a psychologist-led internet-based CBT intervention highlights this issue [37]. The daily delivery of internet-based CBT demonstrated a more substantial reduction in BMI compared to the weekly sessions conducted by nutritionists, suggesting that the interaction frequency rather than the professional role might influence outcomes. Nonetheless, neither approach significantly impacted participants’ stress, anxiety, or depression levels [35]. Furthermore, low adherence to tools such as food diaries, perceived as the “least helpful component” [42], indicates that such tools may trigger feelings of shame or self-criticism among users. Future research should explore the integration of 1-on-1 technical assistance and peer-group problem-solving to address these challenges [38], emphasizing the need for personalization, improved communication, empathetic care, and the integration of multidisciplinary care teams to address both physical and mental health issues in digital health care settings.

The studies collectively revealed certain key themes: misunderstanding and conflicting messages, negative evaluations and weight stigma, the need for a compassionate and empathetic approach, and confusion about HP roles and responsibilities in the context of obesity and mental health care (Table 3). These themes, along with detailed narratives, benefits, and limitations associated with obesity and mental health communication derived from the 8 included studies, are documented in Multimedia Appendix 4. This compilation aligns with the biopsychosocial theoretical model developed by Engel [32], illustrating the integrated approach needed to address these issues effectively.

## Discussion

### Overview

**Figure 3:**
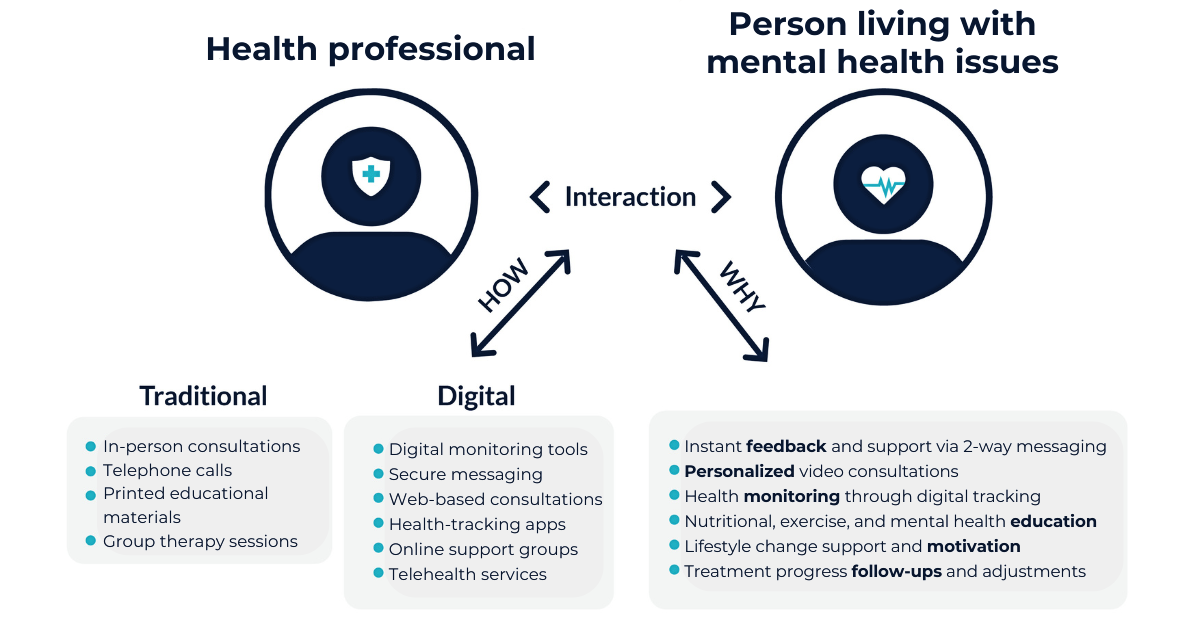
The role of digital technologies in supporting bidirectional communication between health professionals and persons living with obesity and mental health issues.

This scoping review reveals a significant research gap in the application of digital technologies for health care communication between HPs and individuals with comorbid obesity and mental health issues [43] (Figure 3). Despite the extensive search strategy, only 8 studies were retrieved for this scoping review, showing the scarcity of published research. Significantly, these papers were published after 2020, highlighting the novelty of the interdisciplinary research focus and emerging interest after the COVID-19 pandemic, which has accelerated the integration of digital technologies into health care settings. This trend is observable across various countries, emphasizing the universal shift toward digital modalities in addressing complex health care needs associated with obesity and mental health, particularly in the postpandemic era.

**Figure 4:**
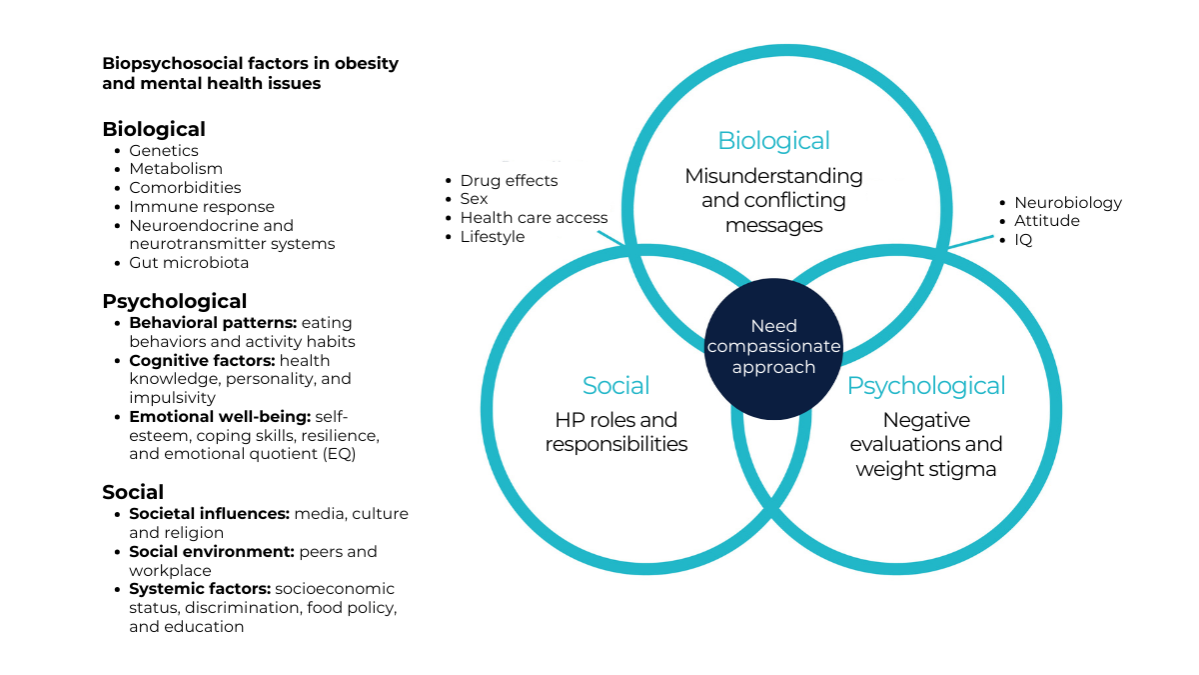
Mapping of the key themes related to obesity and mental health complexities from the 8 included studies [35-42] to the biopsychosocial theoretical model. EQ: emotional quotient; HP: health professional.

Although prior studies have explored the use of technology in the realms of obesity [17,44,45], no research has yet investigated its role in managing comorbid obesity and mental health communication. This review suggests the necessity for more primary research to evaluate whether digital technologies can enhance health communication practices, improve care, and engage users; however, it also highlights a lack of existing studies addressing the combined challenges of obesity and mental health issues. Further research should aim to understand how technological advances can support communication between HPs and individuals living with obesity and mental health issues to deliver person-centered care that meets the biopsychosocial needs of this population [20]. Figure 4 [35-42] portrays the complexities of these biopsychosocial factors and the mapping of the key research themes from the 8 included studies to the biopsychosocial theoretical model.

### Uses of Digital Technologies in Health Care Communication Among Those With Obesity and Mental Health Issues

Although research in this area remains limited, the available evidence suggests that digital platforms have a growing role in bridging the communication gap between HPs and clients, especially in a post–COVID-19 era marked by disruptions in physical access to HPs [46]. Digital platforms, including those providing web-based interfaces, social media networks, and synchronous video call features (Figure 3), facilitate services such as virtual appointments and health monitoring across different professional domains. The findings complement the World Health Organization report [47], highlighting that tele–mental health has emerged as an effective medium for delivering education and psychological treatments, offering an alternative or supplementary approach to traditional therapies [46].

However, this review reveals variability in participant preferences for intervention delivery, highlighting the potential benefits of combining regular HP visits with extended mobile health coaching [35,40,41]. In terms of the frequency and scheduling of HP sessions, some participants found that weekly scheduled telephone check-ins were helpful [35,42], whereas others wanted more flexibility around the competing demands of family or work commitments [35,36,42]. Such clarity is vital for ensuring a comprehensive and unified approach to treating obesity and mental health issues, supporting a more cohesive and integrated care experience for participants.

### Benefits of Digital Technologies in Obesity and Mental Health Communication

Digital technologies enhance obesity and mental health care by offering web-based counseling, personalized care, and tailored interventions [35,41], facilitating a more effective response to individual needs. This personalized approach is significant in ensuring that each individual’s unique health journey is adequately considered and managed (Multimedia Appendix 4). A key element in the success of digital health care interventions is the formation of a therapeutic alliance within web-based settings. The “Meet Your Mentor” program exemplifies the advantages of digital health care, providing a platform for clients to share their stories in a private, in-depth, initial, 1-on-1, 1.5-hour consultation [35]. This indicates the value of individualized attention in digital interventions to facilitate a deeper connection and understanding between the client and the provider.

The shift toward digital health care platforms has significantly reduced the necessity for in-person interactions, mitigating the stigma and negative perceptions often linked to certain health conditions [48]. This change has enhanced client engagement by alleviating the discomfort of activities such as physical weigh-ins, which are more daunting in a group setting [35]. Digital modalities provide anonymity, supporting an environment that encourages open and honest communication, essential for overcoming the challenges of the stigma prevalent in traditional settings [48]. Prior studies support the effectiveness of digital health care in promoting better client-provider interactions through the privacy and detachment it offers compared to face-to-face consultations [35,49].

Furthermore, digital platforms support social learning and compassionate care through peer-to-peer interactions [36,37], promoting mutual support and understanding among participants [50]. Digital technologies have been effectively used to maintain connectivity and engagement between participants, with higher adherence observed in interventions that were tailored to unique individual needs [35,40]. A recent scoping review identified the need to explore effective communication techniques that could be adopted to maintain interpersonal client-provider connections in digitally enabled health care environments [49]; however, the evidence is still unclear, and no studies focus on populations with obesity and mental health issues. This review also identifies a need for more comprehensive interventions to simultaneously address the complex challenges posed by comorbid mental health issues and obesity.

### Limitations of Digital Technologies in Obesity and Mental Health Communication

Despite the potential of digital technology use for health care communication in the context of obesity and mental health issues, there are certain limitations, as this review reveals. Variability in adherence to digital interventions suggests that not all components are equally effective across different settings. The COVID-19 pandemic restrictions further highlighted engagement and retention issues [36,37], showing the need for adaptable research designs to accommodate unpredictable circumstances [36,41]. Issues such as the inconsistent use of self-reported food diaries [40,42] point to the need for interventions that consider individual preferences and behaviors (Table 6). Nicol et al [41] reported the need for more comprehensive symptom assessments, while Aschbrenner et al [36] noted poor adherence, suggesting that young adults prefer individually tailored mobile health coaching over group-based sessions. The variability in engagement and adherence, especially in group-delivered sessions, points to a need for more customized approaches that consider individual preferences and lifestyles.

The current body of evidence identifies a gap in addressing the social complexities associated with comorbid obesity and mental health issues. The studies included in this review lack sufficient detail on communication preferences within interventions [38], highlighting the need for research into effective, compassionate communication strategies to achieve person-centered care [49]. The complexity of various HP roles involved in implementing digital technologies, compounded by conflicting messages and weight stigma [44,51,52], highlights the necessity for empathy and an understanding of the unique challenges faced by individuals with comorbid conditions. Recent research has explored compassionate digital health care [49] but not specifically in the context of obesity and mental health issues.

Future research should focus on tailoring digital interventions to meet the mental health needs of those with comorbid conditions, enhancing personalization, developing engagement strategies, incorporating compassionate care approaches, and defining optimal roles for HPs in digital settings (Table 6). These areas represent critical steps toward improving the effectiveness of digital health care interventions for individuals facing the dual challenges of obesity and mental health issues.

### Interdisciplinary and Person-Centered Care

The integration of HP disciplines and the use of diverse digital technologies in interventions (Table 3) align with the biopsychosocial model of health [32], offering holistic care for those with comorbid obesity and mental health issues. Government initiatives, such as the UK Department of Health’s Mental Health Strategy 2021-2031, support digital, multidisciplinary, person-centered care [53-55]; yet, organizational barriers often prevent effective care delivery [39]. This review identified a potential disconnect between policy recommendations and their practical implementation in health care settings [39], while the recollection of weight stigma experiences, known to harm mental and physical health, underscores the need for action at the policy level [51].

This review points to a lack of technology acceptance theoretical models in current research [4], suggesting a need for frameworks, such as the Unified Theory of Acceptance and Use of Technology 2 formulated by Venkatesh et al [56], to understand the adoption of digital technology in obesity and mental health care. The studies included in this review did not use advanced technologies such as artificial intelligence and virtual reality (VR) [53,57,58], which could offer personalized immersive interventions and create new avenues for engagement. Innovative treatment modalities such as exposure therapy in VR [59] and detailed behavioral data collection through digital phenotyping [57] could help better understand and treat mental health conditions. This disparity suggests a gap in using emerging technologies to enhance the quality and reach of digitally supported health care services [53].

The interdisciplinary nature of interventions aimed at improving physical and mental health among individuals with SMI is evident in the collaborative efforts across various disciplines, including mental health services, fitness professionals, and digital technology experts [35,36,38-40]. Programs such as InSHAPE and PeerFIT demonstrate the benefits of community-based partnerships and professional diversity [36,38] by providing health-mentoring sessions at local community fitness facilities. Integrating individuals with SMI into the broader community promotes social inclusion and reduces stigma. However, this review also points to a deficiency in compassionate communication through digital health care, emphasizing the necessity for strategies that support empathy and understanding in treating obesity and mental health issues.

The PeerFIT and Weight Change for People With Serious Mental Illness interventions illustrate the value of interdisciplinary collaboration, engaging professionals from various backgrounds, such as mental health, fitness, psychology, and social work, trained in health coaching [35,36]. These initiatives, alongside the stakeholder engagement strategy proposed by Haddad et al [39] show the effectiveness of integrating diverse expertise and community involvement in health interventions. Such approaches ensure that the interventions are responsive to the real-world needs of individuals with SMI, leveraging digital technology for more accessible, inclusive health care solutions. This emphasizes the need for interdisciplinary and person-centered principles in developing next-generation communication tools, aiming to comprehensively address the multifaceted needs of those with comorbid conditions (Table 7).

Recent research has acknowledged that technology use influences how HPs perform their work and interact with clients [49]; however, little is known about how compassion is conveyed through digital health care modalities, especially in the context of obesity and mental health care. Despite the widespread adoption of digital health technologies and their potential to impact client outcomes positively [49,55,57,60], strategies for compassionate digital communication for complex health issues are underexplored, signaling a need for research on compassionate, digital approaches for obesity and mental health support (Table 7).

This review emphasizes the need for sociocultural sensitivity in tailoring messages and goals in digital interventions. While most of the included studies reported improvements in physical health outcomes such as weight and BMI, the lack of significant progress in mental health outcomes was a significant finding. This discrepancy raises questions about the comprehensiveness of these interventions in addressing the dual challenges of obesity and mental health issues [42]. Individuals with mental health issues may have trouble engaging with routine WM interventions, and bespoke options are rarely offered in routine practice [35].

**Table 7:** Proposed requirements for next-generation communication tools based on the findings from the reviewed studies (n=8).

Requirement	Description	Justification
Comprehensive care	Content addressing both physical and mental health needs, including modules for digital CBT^a^, stress management, dietary tracking, and physical activity monitoring with real-time feedback and support	Ensures holistic care by addressing the intertwined nature of physical and mental health, improving overall well-being
Personalization and tailoring	Customized content and dynamic intervention adjustments using AI^b^ and machine learning based on user data (eg, behavior, cognition, and emotion)	Personalization and feedback based on individual user data (eg, behavior, cognition, and emotion) have been shown to improve engagement and adherence [39,40]
Enhanced engagement and adherence	Engaging interfaces, gamification, reminders, incentives, and progress-tracking features	Increases user motivation and sustained tool use, crucial for long-term health management
Compassionate design	Features promoting empathy, including personalized messaging, web-based support groups, videoconferencing, secure messaging, and forums for peer support Training for HPs^c^ on compassionate care delivery	A compassionate approach addresses psychological and social needs in health care, supporting a sense of community and belonging [35,36,38,42]
Multidisciplinary support	Tools for HPs to monitor progress, provide feedback, and collaborate across disciplines	Enhances care fidelity and patient retention through comprehensive, coordinated care approaches [38,39,42]
Stigma reduction	Anonymous participation options and educational content to counteract stereotypes and misinformation	Reduces barriers to seeking help by mitigating stigma and promoting a more inclusive care environment
Anonymity and privacy	Ensuring user anonymity and privacy to reduce fears of judgment and stigma	Encourages wider participation by providing a secure and confidential user experience. Anonymity encourages participation by mitigating concerns about judgment [35,37]
Integration with existing health care systems	Compatibility with health care platforms for streamlined care coordination. Adaptability across health care settings and comprehensive training for users and providers	Seamless integration with health care systems can enhance the efficacy and sustainability of digital interventions [37,38] Compliance with health care regulations and data protection standards
Technical assistance	Continuous support and education for users and providers to maximize digital tool benefits and adapt to updates	Ensures that users and providers can effectively use the full range of tool capabilities, enhancing overall intervention success

^a^CBT: cognitive behavioral therapy.

^b^AI: artificial intelligence.

^c^HP: health professional.

Digital technologies play a vital role in extending health care reach, especially to communities considered marginalized, by providing anonymous, unbiased access to services, thus reducing pressure on health care systems [60]. They serve as an accessible bridge for support while individuals await further assistance [57,60]. However, achieving their full potential requires ongoing innovation, tailored designs, and an inclusive and interdisciplinary strategy that equally addresses physical and mental health [12,18,58,61].

To address these gaps, future research should explore more extensive interventions with larger participant groups and extended follow-up assessments to gauge the sustained impacts of digital interventions. Customizing digital tools to fit individual needs, aligned with policy support, is essential [47,54]. These tools should facilitate deeper engagement with the complex needs of those facing both obesity and mental health challenges, moving beyond mere information dissemination. High-quality data use could improve direct client interactions [57], although the included studies lack detailed insights into the specific characteristics and preferences of this demographic, highlighting a gap in effective digital communication strategies for HPs catering to individuals with comorbid obesity and mental health issues.

### Limitations

Study limitations included the small sample of 8 selected studies, with limited sample sizes (ranging from 17 to 150 participants), which may limit the generalizability of the findings. A common reliance on self-reported data, such as food diaries, introduces susceptibility to inaccuracies and social desirability bias. Of the 8 included studies, 7 reported intervention durations. Most (6/7, 86%) were of short duration (≤12 weeks) and lacked long-term follow-ups, limiting insights into the sustainability of outcomes. One study did not specify its duration, highlighting the need for standardized reporting. The interventions noted feasibility challenges related to the labor intensity required from HPs and implementation costs [38]. The predominance of female participants and varied study designs, including RCTs and feasibility studies, offer varied perspectives but also raise questions about the broader applicability of the results.

Digital interventions, while beneficial, risk exacerbating social isolation or excluding certain populations due to reduced physical contact and digital accessibility issues, such as a lack of digital skills, limited resources, or unwillingness to engage with digital platforms. There was a lack of exploration of participant preferences for intervention format, which is crucial for tailoring interventions to individual needs and enhancing engagement and effectiveness. This highlights a need to consider digital inclusion in future digital health initiatives.

Methodologically, inconsistencies in the inclusion criteria for SMI and the measurement of mental health outcomes were noted (Table 2). This ambiguity hinders the ability to definitively assess the impact of digital interventions on mental health issues, pointing to the need for more standardized criteria in future research. In addition, the focus on diagnostic criteria may limit the understanding of underlying processes common across weight and mental disorders, suggesting a shift toward a more process-oriented approach in future studies [19].

### Conclusions

This scoping review sheds light on the evolving role of digital technologies in health care communication for individuals living with obesity and mental health issues. It reveals the use of diverse modalities to bridge communication gaps; deliver education, motivation, and counseling; and enhance care accessibility and multidisciplinary support. However, it also highlights limitations related to inconsistent adherence, challenges in implementation, and the absence of technology acceptance theoretical models in current research. A key finding is the tangible improvement in physical health outcomes in obesity management through digital means. However, the effectiveness of these technologies in concurrently addressing mental health issues is less conclusive, marking a critical area for further investigation. The limited but insightful studies show the interdisciplinary and evolving nature of this field, accelerated by recent advancements in digital health care delivery.

The findings advocate for future research to be more extensive and inclusive, with an emphasis on the long-term impacts of digital interventions. This includes conducting follow-up assessments to understand the sustained effects and integrating innovative technologies such as artificial intelligence, VR, and machine learning to enrich mental health interventions [53,59]. The application of frameworks such as the Unified Theory of Acceptance and Use of Technology 2 model [56] could provide valuable insights into the adoption and use of digital technology by HPs in addressing complex health issues. While digital tools offer promise for enhancing health care communication in obesity management, their full potential in addressing concurrent mental health issues is yet to be realized. The review calls for custom digital solutions that address the biopsychosocial needs of those facing both obesity and mental health challenges, advocating for an interdisciplinary approach that encompasses various HP roles and comprehensive nutrition support [40]. This research is pivotal for advancing clinical practices and informing health policy, emphasizing the ongoing need for innovation and adaptation in digital health care communication strategies.

In conclusion, the journey toward using the full potential of digital technologies in health care communication is ongoing, with enhanced accessibility, reduced stigma, and personalized care as key benefits. Nevertheless, there remains a need to develop more comprehensive and integrated digital solutions to effectively tackle the complex interplay of obesity and mental health issues [36]. The vital steps for moving forward include addressing participant preferences; encouraging collaborative design [57]; and ensuring that interventions provide consistency, cohesion, and choice for participants [54].

## Appendix

Multimedia Appendix 1 PRISMA-ScR (Preferred Reporting Items for Systematic Reviews and Meta-Analyses extension for Scoping Reviews) checklist.

Multimedia Appendix 2 Search strategy.

Multimedia Appendix 3 Complete search strategy and database results.

Multimedia Appendix 4 Table of results mapping biopsychosocial constructs to the research themes.

## Abbreviations

CBT: cognitive behavioral therapy
HP: health professional
PRISMA-ScR: Preferred Reporting Items for Systematic Reviews and Meta-Analyses extension for Scoping Reviews
RCT: randomized controlled trial
ROB: risk of bias
SMI: serious mental illness
VLC: virtual learning collaborative
VR: virtual reality
WM: weight management

## Data Availability

All data generated or analyzed during this study are included in this published paper (and its supplementary information files).
