# Supplementary material for: Assessing the Uses, Benefits, and Limitations of Digital Technologies Used by Health Professionals in Supporting Obesity and Mental Health Communication: Scoping Review
Source: J Med Internet Res. 2025 Feb 10;27:e58434. doi: 10.2196/58434 (PMC11851038; doi:10.2196/58434)
Supplement: Multimedia Appendix 2 [file jmir_v27i1e58434_app2.docx]

**Multimedia Appendix 2: Search strategy**

|  | **MESH** | **Keywords** | **Search String** |
| --- | --- | --- | --- |
| Population | Health Personnel  **MeSH HEADING:** HEALTH PERSONNEL  **SCOPE:** Men and women working in the provision of health services, whether as individual practitioners or employees of health institutions and programs, whether or not professionally trained, and whether or not subject to public regulation. (From A Discursive Dictionary of Health Care, 1976)  **Used For:**   - - health care professional   - health care professionals   - health care provider   - health care providers   - health personnel   - healthcare worker   - healthcare workers   - personnel, health   - professional, health care   - provider, health care   **CINAHL Scope Health Personnel**  Individuals who provide health services.  Plus Mesh Headings:  Alternative Health Personnel  Allied Health Personnel  Mental Health Personnel  Multiskilled Health Practitioners | Health Professional;  Health Practitioner;  Health worker;  Healthcare Professional;  Clinician;  Medical Practitioner;  Doctor;  Public Health;  GP; General Practitioner;  Physician;  Surgeon;  Paediatrician;  Psychiatrist;  Radiologist.  Nurse;  Midwife.  Arts therapist;  Recreational Therapist;  Dance & Movement Therapist.  Biomedical scientist  Chiropodist; Podiatrist  Clinical scientist  Dietitian; Dietician;  Hearing aid dispenser  Occupational therapist  Operating department practitioner  Orthoptist  Paramedic  Physiotherapist; Physical therapist;  Psychologist; Prosthetists / orthotist; Radiographer; Speech and language therapist; Speech therapist; Audiologist.  Dentist; Dental nurse; Pharmacist; Dispensing chemist  Optometrist; Optician;  Nutritionist.  Complementary Therapist. | **HEALTH PERSONNEL (subject heading)**  Keyword n = 42 derived from ISCO, 2008.  **Ovid Medline search:**  Health* professional or Health Practitioner or Health* worker or Clinician or Medical Practitioner or Doctor or Public Health or GP or General Practitioner or Physician or Surgeon or P#ediatric* or Psychiatrist or Radiologist or Nurs* or Midwife or Biomedical scientist or Chiropodist or Podiatrist or Clinical scientist or Dieti#ian or Hearing aid dispenser or Occupational or Operating department or Orthoptist or Paramedic or Physi* therap* or psychologist or Psychotherap or Prosthetist or orthotist or Radiographer or Speech and language therapist or Speech therap* or Audiologist or Art* therapist or Drama therapist or Music therapist or Dentist or Pharmacist or Nutritionist or Complementary therapist  **Scopus Database search:**  “Health* professional” or “Health Practitioner” or “Health* worker” or Clinician or “Medical Practitioner” or Doctor or “Public Health” or GP or “General Practitioner” or Physician or Surgeon or P#ediatric* or Psychiatrist or Radiologist or Nurs* or Midwife or “Biomedical scientist” or Chiropodist or Podiatrist or “Clinical scientist” or Dieti#ian or “Hearing aid dispenser” or Occupational or “Operating department” or Orthoptist or Paramedic or “Physi* therap*” or psychologist or Psychotherap or Prosthetist or orthotist or Radiographer or “Speech and language therapist” or “Speech therap*” or Audiologist or “Art* therapist” or “Drama therapist” or “Music therapist” or Dentist or Pharmacist or Nutritionist or “Complementary therapist”  **IEEE Xplore limited to 25 consecutive search terms**  “Health* professional” or “Health Practitioner” or “Health* worker” or Clinician or “Medical Practitioner” or Doctor or “Public Health” or Physician or Surgeon or P#ediatric* or Psychiatrist or Nurs* or Midwife or Dieti#ian or Occupational or “Physi* therap*” or psychologist or Psychotherap or “Speech therap*” or “Art* therapist” |
| Concept | Digital Technology Intervention  **MeSH HEADING:** DIGITAL TECHNOLOGY  **SCOPE:** The design and development of devices and procedures that collect, store, analyze, manipulate, and display numerically encoded information, usually via processes encoded in the binary number system.  **Used For:**   - - digital electronics   - digital technologies   - digital technology   - electronics, digital   - technologies, digital   - technology, digital | Technology,  digital health,  mobile, mhealth,  e-mental, M-HEALTH,  E-HEALTH, ehealth,  online intervention,  online, virtual, remote,  e-therapy,  Digital therapeutics,  Digital communication,  genomic testing, genotyping,  digital medicine,  precision medicine,  health analytics, data analytics,  artificial intelligence, AI,  robotics, big data,  Internet of Things, IoT,  blockchain,  digital electronics, EHR,  electronic health record,  digital record, patient portal,  messaging, telecare,  TELE-HEALTH, telehealth,  telemedicine,  video consultation,  smartphone, application,  medical app, wellness app,  fitness app,  social media,  biosensor, wearable,  speech recognition,  voice recognition,  machine learning, automated image interpretation, neuroimaging,  virtual reality, VR, augmented reality,  gaming, serious games,  simulation. | **DIGITAL TECHNOLOGY (subject heading)**  Keyword n = 71 derived from Topol. E., 2019.  ***Ovid Medline string***  technolog* or digital or online or virtual or remote or web-based or mobile or mhealth or e-mental or E-HEALTH or M-HEALTH or online intervention or remote intervention or virtual intervention or web-based intervention or internet-based therap* or internet-based treatment or internet-based intervention or digital intervention or ehealth or computer assist* or telemonitor* or Digital therapeutics or e-therapy or Digital comm* or telecommunication* or gen* testing or genotyping or health analytic* or data analytic* or precision medicine or telecare or telehealth or TELE-HEALTH or telemedicine or video consult* or econsult* or teleconsult* virtual consult* or digital medicine or artificial intelligence or AI or robotics or digital electronics or big data or Internet of Things or IoT or blockchain or electronic health record or digital record or patient portal or messaging or smartphone or social media or application or medical app or wellness app or fitness app or biosensor or wearable or speech recognition or voice recognition or machine learning or automated image interpretation or neuroimaging or virtual reality or VR or augmented reality or gaming or serious games or simulation  ***Scopus Database Search:***  "digital technolog*" or technolog* or digital or online or virtual or remote or "web-based" or mobile or mhealth or "e-mental" or "E-HEALTH" or "M-HEALTH" or "online intervention" or "remote intervention" or "virtual intervention" or "web-based intervention" or "internet-based therap*" or "internet-based treatment" or "internet-based intervention" or "digital intervention" or ehealth or "computer assist*" or telemonitor* or "Digital therapeutics" or "e-therapy" or "Digital comm*" or telecommunication* or “gen* testing” or genotyping or "health analytic*" or "data analytic*" or "precision medicine" or telecare or telehealth or "TELE-HEALTH" or telemedicine or "video consult*" or econsult* or teleconsult* or "virtual consult*" or "digital medicine" or "artificial intelligence" or AI or robotics or "digital electronics" or "big data" or "Internet of Things" or IoT or blockchain or "electronic health record" or "digital record" or "patient portal" or messaging or smartphone or "social media" or application or "medical app" or "wellness app" or "fitness app" or biosensor or wearable or "speech recognition" or "voice recognition" or "machine learning" or "automated image interpretation" or "neuroimaging" or "virtual reality" or VR or "augmented reality" or gaming or "serious games" or simulation |
| Context | Obesity  **MeSH HEADING:** OBESITY  **SCOPE:** A status with BODY WEIGHT that is grossly above the recommended standards, usually due to accumulation of excess FATS in the body. The standards may vary with age, sex, genetic or cultural background. In the BODY MASS INDEX, a BMI greater than 30.0 kg/m2 is considered obese, and a BMI greater than 40.0 kg/m2 is considered morbidly obese (MORBID OBESITY).  **NOTE:** obese hyperglycemic mice are indexed MICE, OBESE: do not add OBESITY or HYPERGLYCEMIA unless disease particularly discussed; /ther consider also OBESITY MANAGEMENT  **REFERENCES:**  **See Related:**   - - APPETITE DEPRESSANTS   - BODY WEIGHT   - DIET, REDUCING   - SKINFOLD THICKNESS   - LIPECTOMY   - ANTI-OBESITY AGENTS   - BARIATRICS - **Used For:**   - obesity   **CINAHL scope Obesity**  An abnormal accumulation of body fat, usually 20% or more over an individual's ideal body weight. Do not use /in infancy and childhood or /in adolescence; prefer precoordinated heading PEDIATRIC OBESITY.  **CINAHL scope Pedatric obesity** An abnormal accumulation of body fat in children or adolescents.  **CINAHL Scope Morbid Obesity**  Condition of weighing two or three, or more, times the ideal weight; so called because it is associated with many serious and life-threatening disorders. | Obese,  obesity,  overweight,  weight management,  weight reduction,  weight loss,  excess body fat,  excess fat,  excess weight,  body mass,  BMI,  metabolic syndrome,  bulimia,  binge eating,  eating disorder,  bariatric surgery | **OBESITY, OBESITY MANAGEMENT (subject heading)**  Keyterm n = 16  **Ovid Medline Search**  obes* or overweight or weight management or weight reduc* or weight loss diet or excess* body fat or excess* weight or excess* fat or body mass or body weight or BMI or metabolic syndrome or bulimia or binge eating or eating disorder or bariatric  **Scopus Database Search:**  obes* or overweight or “weight management” or “weight reduc*” or “weight loss diet” or “excess* body fat” or “excess* weight” or “excess* fat” or “body mass” or “body weight” or BMI or “metabolic syndrome” or bulimia or “binge eating” or “eating disorder” or bariatric |
|  | Mental Health  **MeSH HEADING:** MENTAL DISORDERS  **SCOPE:** Psychiatric illness or diseases manifested by breakdowns in the adaptational process expressed primarily as abnormalities of thought, feeling, and behavior producing either distress or impairment of function.  **Used For:**   - - behavior disorders   - diagnosis, psychiatric   - illness, mental   - mental disorder   - mental disorder, severe   - mental disorders   - mental disorders, severe   - mental illness   - mental illnesses   - psychiatric diagnosis   - psychiatric disease   - psychiatric diseases   - psychiatric disorder   - psychiatric disorders   - psychiatric illness   - psychiatric illnesses   - severe mental disorder   - severe mental disorders | Mood,  Stress,  Mental health,  mental illness,  mental ill health,  Depression,  Anxiety,  Burnout,  Post-Traumatic Stress Disorder,  PTSD,  Panic disorder,  Phobia,  Psychotic,  Psychosis,  Psychiatric illness,  Schizophrenia,  Addiction,  Eating disorder,  Bipolar disorder,  Severe Mental Illness. | **MENTAL DISORDER (subject heading)**  Keyword n = 15  **Medline Search**  Mood or Stress or Mental ill* or Depressi* or Anxiety or Burnout or Post*Traumatic Stress or PTSD or Panic or Phobia or Psycho* or Psychiatric or Schizophreni* or Addict* or Bipolar  **Scopus Database Search**  Mood or Stress or “Mental ill*” or Depressi* or Anxiety or burnout or “Post*Traumatic Stress” or PTSD or Panic or Phobia or Psycho* or psychiatric or Schizophreni* or Addict* or Bipolar |
