# Supplementary material for: Assessing the Uses, Benefits, and Limitations of Digital Technologies Used by Health Professionals in Supporting Obesity and Mental Health Communication: Scoping Review
Source: J Med Internet Res. 2025 Feb 10;27:e58434. doi: 10.2196/58434 (PMC11851038; doi:10.2196/58434)
Supplement: Multimedia Appendix 3 [file jmir_v27i1e58434_app3.docx]

**Multimedia Appendix 3: Complete search strategy and database results.**

| **Appendix 3a: Search Strategy for Ovid MEDLINE(R) ALL <1946 to April 11, 2023>** | |
| --- | --- |
| **Heading #** | **Search statement** |
| 1 | Health Personnel/ 62672 |
| 2 | (((Health* professional or Health Practitioner or Health* worker or Clinician or Medical Practitioner or Doctor or Public Health or GP or General Practitioner or Physician or Surgeon or P#ediatric* or Psychiatrist or Radiologist or Nurs* or Midwife or Biomedical scientist or Chiropodist or Podiatrist or Clinical scientist or Dieti#ian or Hearing aid dispenser or Occupational or Operating department or Orthoptist or Paramedic or Physi* therap* or psychologist or Psychotherap or Prosthetist or orthotist or Radiographer or Speech) and language therapist) or Speech therap* or Audiologist or Art* therapist or Drama therapist or Music therapist or Dentist or Pharmacist or Nutritionist or Complementary therapist).mp. [mp=title, book title, abstract, original title, name of substance word, subject heading word, floating sub-heading word, keyword heading word, organism supplementary concept word, protocol supplementary concept word, rare disease supplementary concept word, unique identifier, synonyms, population supplementary concept word, anatomy supplementary concept word] 57687 |
| 3 | 1 or 2 119948 |
| 4 | Digital Technology/ 647 |
| 5 | (technolog* or digital or online or virtual or remote or web-based or mobile or mhealth or e-mental or E-HEALTH or M-HEALTH or online intervention or remote intervention or virtual intervention or web-based intervention or internet-based therap* or internet-based treatment or internet-based intervention or digital intervention or ehealth or computer assist* or telemonitor* or Digital therapeutics or e-therapy or Digital comm* or telecommunication*or gen* testing or genotyping or health analytic* or data analytic* or precision medicine or telecare or telehealth or TELE-HEALTH or telemedicine or video consult* or econsult* or teleconsult* or virtual consult* or digital medicine or artificial intelligence or AI or robotics or digital electronics or big data or Internet of Things or IoT or blockchain or electronic health record or digital record or patient portal or messaging or smartphone or social media or application or medical app or wellness app or fitness app or biosensor or wearable or speech recognition or voice recognition or machine learning or automated image interpretation or neuroimaging or virtual reality or VR or augmented reality or gaming or serious games or simulation).mp. [mp=title, book title, abstract, original title, name of substance word, subject heading word, floating sub-heading word, keyword heading word, organism supplementary concept word, protocol supplementary concept word, rare disease supplementary concept word, unique identifier, synonyms, population supplementary concept word, anatomy supplementary concept word] 3276045 |
| 6 | 4 or 5 3276045 |
| 7 | Obesity, Morbid/ or Pediatric Obesity/ or Obesity/ or Obesity Management/ 249798 |
| 8 | (obes* or overweight or weight management or weight reduc* or weight loss diet or excess* body fat or excess* weight or excess* fat or body mass or body weight or BMI or metabolic syndrome or bulimia or binge eat* or eating disorder or bariatric).mp. [mp=title, book title, abstract, original title, name of substance word, subject heading word, floating sub-heading word, keyword heading word, organism supplementary concept word, protocol supplementary concept word, rare disease supplementary concept word, unique identifier, synonyms, population supplementary concept word, anatomy supplementary concept word] 1037358 |
| 9 | 7 or 8 1037358 |
| 10 | exp Mental Disorders/ 1421433 |
| 11 | (Mood or Stress or Mental ill* or Depressi* or Anxiety or burnout or Post*Traumatic Stress or PTSD or Panic or Phobia or Psycho* or psychiatric or Schizophreni* or Addict* or Bipolar).mp. [mp=title, book title, abstract, original title, name of substance word, subject heading word, floating sub-heading word, keyword heading word, organism supplementary concept word, protocol supplementary concept word, rare disease supplementary concept word, unique identifier, synonyms, population supplementary concept word, anatomy supplementary concept word] 3565814 |
| 12 | 10 or 11 4098746 |
| 13 | 9 and 12 171248 |
| 14 | 3 and 6 and 13 98 |
| 15 | limit 14 to humans 87 |

Appendix 3b: Database Search Results

| **Appendix 3b: Boolean search term results** | | | |  | **DB 1** | **DB 2** | **DB 3** | **DB 4** | **DB 5** | **DB 6** | **DB 7** | **DB 8** |  | **TOTAL n** |
| --- | --- | --- | --- | --- | --- | --- | --- | --- | --- | --- | --- | --- | --- | --- |
| **PCC** |  | **Subject Heading** | **Search terms** | **Boolean connector** | **Medline Ovid** | **CINAHL ultimate** | **Scopus** | **Science Direct** | **PsycINFO Ovid** | **IEEE Explore Digital Library** | **ACM Digital Library** | **ClinicalTrials.gov** | **Citation searching** |  |
| **POPULATION** | 1 | **Health personnel** | Health Personnel/ |  | 62672 | 63,553 |  |  | 188406 | 889 | 14598 |  |  |  |
|  | 2 |  | keywords as per comment |  | 57687 | 1,094,889 | 4,168,897 | 23534 | 10256 | 2,572 |  |  |  |  |
|  | 3 | **1 OR 2** |  | OR | 119948 | 1,131,421 |  |  | 196734 | 3,401 | 22,011 |  |  |  |
| **CONCEPT** | 4 | **Digital Technology** | Digital Technology/ |  | 647 | 1,993 |  |  | 164524 | 45791 | 91,562 |  |  |  |
|  | 5 |  | keywords as per comment |  | 3276045 | 527,976 | 19,280,250 | 725582 | 629960 |  |  |  |  |  |
|  | 6 | **4 or 5** |  | OR | 3276045 | 528,608 |  |  | 657103 |  |  |  |  |  |
| **CONTEXT** | 7 | **Obesity** | Obesity, Morbid/ or Pediatric Obesity/ or Obesity/ |  | 249798 | 115,602 |  |  | 28315 | 1021 | 276 |  |  |  |
|  | 8 |  | keywords as per comment |  | 1037358 | 230,295 | 1,629,597 | 402091 | 119400 |  | 14411 |  |  |  |
|  | 9 | **7 or 8** |  |  | 1037358 | 277,724 |  |  | 119400 |  |  |  |  |  |
|  | 10 | **Mental disorder** | Mental Disorder |  | 1421433 | 68,539 |  |  | 1016406 | 1,270 | 5,336 |  |  |  |
|  | 11 |  | keywords as per comment |  | 3565814 | 601,664 | 6,692,916 | 610766 | 2256124 |  | 6801 |  |  |  |
|  | 12 | **10 or 11** |  | OR | 4098746 | 638,739 |  |  | 2571322 |  |  |  |  |  |
|  | 13 | **9 AND 12** |  |  | 171248 | 33,243 | 267,506 |  | 68129 |  |  |  |  |  |
|  | 14 | **3 and 6 and 13** | HP + Tech + comorbid Obesity MH = one string | AND | 98 | 668 | 1,577 | 357894 | 141 | 1442 | 15 |  |  |  |
|  | *15* | **"limit 13 to (human)"** | limit 14 to humans | AND' 'Human' | *87* | *408* | 817 | 26 | *136* | *156* | *15* | *7* | *4* | *1656* |
