# Supplementary material for: Assessing the Uses, Benefits, and Limitations of Digital Technologies Used by Health Professionals in Supporting Obesity and Mental Health Communication: Scoping Review
Source: J Med Internet Res. 2025 Feb 10;27:e58434. doi: 10.2196/58434 (PMC11851038; doi:10.2196/58434)
Supplement: Multimedia Appendix 4 [file jmir_v27i1e58434_app4.docx]

**Multimedia Appendix 4: Table of results mapping biopsychosocial constructs to the research themes.**

**Integration of Biopsychosocial constructs in obesity and mental healthcare communication: Key research themes, benefits, and limitations in 8 included studies.**

| **Key research themes and papers** | **Biopsychosocial (BPS) construct and related study narratives** | **Benefits** | **Limitations** |
| --- | --- | --- | --- |
| **Misunderstanding and conflicting messages**    [^1-2, 4-5, 7-8^] | *B:* "Continuous changes in nutrition training", need for adaptable strategies.  *P:* Misunderstandings of SMS prompts and nutrition information affect intervention engagement.  *S:* Cultural differences in the context of Covid "rumored that obesity boosts the immune system" impacting perceptions. | Personalised digital CBT and tailored feedback.  - High adherence and motivation.  - Digital CBT tailored to individual characteristics (behaviour, cognition, emotion, motivation, and physical domains). | Engagement barriers related to symptom severity.  - Need for tailored interventions to clinical population and setting.  - Need for research on genetic factors and behaviour patterns. |
| **Negative evaluations and weight stigma**    [^1, 2, 3, 5, 6^] | *P:* Experiences of "shame/avoidance, low self-esteem and negative self-evaluations".  *S:* social anxiety related to being weighed in public and societal judgements: "I have no intention of being weighed in front of others or (a)nother. I know I am overweight and it causes me great distress and anxiety. I do not want to add to this. It is embarrassing to be weighed. A judgement is being made". | Anonymity in ICBT;  - reduces fear of negative evaluations, encouraging participation.  Online preference to avoid stigma:  "removed the fear of social situations and getting weighed or judged in front of others". | Technical assistance needs;  - lack of preference data as social challenges in implementation and communication approach. |
| **HP roles and responsibilities**    [^2-5, 7^] | *S:* Emphasis on "multidisciplinary team training" and the role of dietitians in obesity CBT:  - "Health mentors and program supervisors were instructed on MI to support persons with SMI in adopting lifestyle changes, including setting personal goals, tracking eating and physical activity behaviors, and understanding the principles of healthy eating and nutrition".  - "Including a Dietitian in the therapeutic process may enhance overall efficacy". | Multidisciplinary virtual collaboration enhances fidelity and retention.  - included nurses, prescribers, community support workers, case managers, psychosocial rehabilitation counsellors and peer supports. | Lacked Nutritionist input in interventions.  - Variations in delivery intensity and professional roles.  - Challenges in integrating healthcare perspectives, training sustainability and programme efficacy. |
| **Need compassionate and empathetic approach**  [^1-2, 4-5, 7-8^] | *B:* Stressors impacting mental health: "we have a way of kind of ignoring things, especially things that make stress bad or make us not feel great".  *P:* Emphasis on effective communication skills: "I think, personally… the empathy, the kind of active listening [the mentor] had going on, I think that was really good".  *S:* Establishing a "therapeutic alliance" beneficial.  Peer support and social learning through peer-to-peer interactions. | Emphasis on mind-body connection.  Greater health promotion impact and reach.  - Ease of online counselling. | Conflicting life demands impacting engagement.  - Significant staff investment required in staff time and associated costs.  - Information on participant preferences would help to tailor future interventions. |
| *Table Key: B = Biological, P = Psychological, S = Social constructs* | | | |

**References for Multimedia Appendix 4**

1. Lee C, Waite F, Piernas C, Aveyard P. Development and initial evaluation of a behavioural intervention to support weight management for people with serious mental illness: an uncontrolled feasibility and acceptability study. *BMC Psychiatry*. 2023;23(1):130. doi:<https://dx.doi.org/10.1186/s12888-023-04517-1>
2. Aschbrenner KA, Naslund JA, Gorin AA, et al. Group Lifestyle Intervention With Mobile Health for Young Adults With Serious Mental Illness: A Randomized Controlled Trial. *Psychiatr Serv*. 2022;73(2):141-148. doi:<https://dx.doi.org/10.1176/appi.ps.202100047>
3. Abedishargh N, Farani AR, Gharraee B, Farahani H. Effectiveness of Internet-based Cognitive Behavioral Therapy in Weight Loss, Stress, Anxiety, and Depression via Virtual Group Therapy. *Iran J Psychiatry Behav Sci*. 2021;15(3). doi:10.5812/IJPBS.113096
4. Bartels SJ, Aschbrenner KA, Pratt SI, et al. Virtual Learning Collaborative Compared to Technical Assistance as a Strategy for Implementing Health Promotion in Routine Mental Health Settings: A Hybrid Type 3 Cluster Randomized Trial. *Adm Policy Ment Health*. 2022;49(6):1031-1046. doi:<https://dx.doi.org/10.1007/s10488-022-01215-0>
5. Haddad R, Badke D’Andrea C, Ricchio A, et al. Using Innovation-Corps (I-Corps™) Methods to Adapt a Mobile Health (mHealth) Obesity Treatment for Community Mental Health Settings. *Front Digit Health*. 2022;4. doi:10.3389/fdgth.2022.835002
6. Kim M, Kim Y, Go Y, et al. Multidimensional cognitive behavioral therapy for obesity applied by psychologists using a digital platform: Open-label randomized controlled trial. *JMIR Mhealth Uhealth*. 2020;8(4). doi:10.2196/14817
7. Nicol G, Jansen M, Haddad R, et al. Use of an Interactive Obesity Treatment Approach in Individuals with Severe Mental Illness: Feasibility, Acceptability, and Proposed Engagement Criteria. *JMIR Form Res*. 2022;6(12). doi:10.2196/38496
8. Yu Z, Roberts B, Snyder J, et al. A Pilot Study of a Videoconferencing-Based Binge Eating Disorder Program in Overweight or Obese Females. *Telemed J E Health*. 2021;27(3):330-340. doi:<https://dx.doi.org/10.1089/tmj.2020.0070>
